# Supplementary material for: Morphological changes in the human mandible associated with the presence of exostoses: A cross-sectional study in two archaeological populations from southern France
Source: PLoS One. 2025 Feb 12;20(2):e0306501. doi: 10.1371/journal.pone.0306501 (PMC11819550; doi:10.1371/journal.pone.0306501)
Supplement: Attachment — Submitted filename: Response to Reviewers.docx [file pone.0306501.s006.docx]

Response to Reviewers

Dear Reviewers,

Thank you for your careful review of our manuscript.

We have considered every one of your comments to improve this article. You will find our responses to each of your suggestions in blue.

Kind regards,

The authors

Reviewer #1: (No Response)

Reviewer #2:

Thank you for the review
paper is improved
all the necessary changes are done
figures and tables meet the paper criteria
thank you

We want to thank you once again for your careful proofreading and the valuable advice you gave us to improve the manuscript.

Reviewer #3: The revision is better but there is a lot of room for improvement. Please improve it more:

- While reporting p-values, please report at least up to 3 decimal places or more, but not fewer.

Thank you for your relevant comment. We've changed that (line 259).

- Besides reporting min, max, mean, and SD, also do report median, Q1, and Q3.

We've added that (line 235).

- Also report 95% Confidence Intervals.

We've added that (line 259).

- Report these parameters for the whole sample as well as both groups.

Thank you for your relevant comment. We've added two tables (lines 240 and 243)

- Attach the statistical raw dataset as supplementary material.

Thank you for your suggestion. The statistical raw dataset has been attached as supplementary material (Appendix S2).

- The paper is still very unorganized. The organization of the paper is done very poorly. The subheadings are the same size as the main headings. You should use different levels of subheadings. Avoid using the font for the main headings for the subheadings.

- Add numbers to the headings and subheadings for better fluency and organization. Like 3, 3.1, 2.3.1...

Thank you for your advice. However, the PLOS ONE manuscript body formatting guidelines, which we have carefully followed, does not mention the addition of numbers. The choice different levels of subheadings has also been calibrated according to PLOS ONE instructions. For these reasons, we have not been able to modify the manuscript according to your suggestions.

[https://journals.plos.org/plosone/s/file?id=wjVg/PLOSOne_formatting_sample_main_body.pdf](https://urldefense.com/v3/__https:/journals.plos.org/plosone/s/file?id=wjVg*PLOSOne_formatting_sample_main_body.pdf__;Lw!!JQ5agg!eiKb1tWKltkYQARehOutVQFJfA1-17cDp0wJdJqVKI2NZgIV4IH1hMQ5PhN1ZDijzWw6CZT-ScEeliHgN0thPpph$)

- The name and details of the statistical software?

These details had been given lines 184-185.
